# The Experience of a Tertiary Referral Center with Endoscopic Management and Combining Percutaneous Intervention for the Treatment of Walled-Off Necrosis: A Stepwise Approach

**DOI:** 10.3390/jcm13164916

**Published:** 2024-08-20

**Authors:** Ali Atay, Ilhami Yuksel

**Affiliations:** 1 Department of Gastroenterology, Ankara Bilkent City Hospital, Ankara 06800, Turkey; yukselilhami@hotmail.com; 2 Department of Gastroenterology, School of Medicine, Ankara Yildirim Beyazit University, Ankara 06800, Turkey

**Keywords:** walled-off necrosis, endoscopic cystogastrostomy, lumen-apposing metallic stent, percutaneous intervention

## Abstract

**Background:** This study aimed to assess the effectiveness and safety of endoscopic management in patients with walled-off necrosis and additionally explore the results of a stepwise approach for combining percutaneous intervention in cases where endoscopic management was inadequate. **Methods:** We included cases of endoscopic management for walled-off necrosis between February 2019 and December 2023. **Results:** Endoscopic management was performed in 11 patients. The median largest dimension was 150 mm. Multiple cavities were present in four patients. Technical success was 90.9%, while clinical success with only endoscopic management was 36.3%. Clinical success could not be achieved with only endoscopic management in patients with a large diameter (≥125 mm) or multi-lobulated walled-off necrosis. Combining percutaneous intervention resulted in success for all patients. Two patients experienced major complications: one suffered from major bleeding, while the other experienced perforation, necessitating surgical intervention. The patient with perforation died due to multi-organ failure. **Conclusions:** Endoscopic management is recommended as the primary treatment method for walled-off necrosis due to its less invasive and higher safety profile. In cases involving large or multi-lobulated walled-off necrosis where clinical success cannot be achieved, combining percutaneous intervention is highly successful and safe. Ultimately, this approach can minimize the need for more invasive surgery.

## 1. Introduction

Walled-off necrosis (WON) is a late complication of acute necrotizing pancreatitis that can lead to permanent organ failure and even death [1,2]. Necrosis in the pancreas develops in approximately 15–20% of all acute pancreatitis (AP) cases, and about 40% of these cases progress to infected or complicated WON [3,4,5,6,7,8]. In the historical process, WON was often managed with surgical methods. However, minimally invasive procedures involving endoscopic interventions, video-assisted retroperitoneal debridement, and percutaneous drainage have become preferred modalities due to their lower morbidity, mortality, cost, and complication rates, as well as providing a better quality of life. Among minimally invasive approaches, endoscopic interventions are the preferred initial method [1,9,10]. In cases of sterile pancreatic necrosis presenting with symptoms such as abdominal pain, nausea, vomiting, and nutritional insufficiency, or in patients with associated complications such as biliary obstruction, recurrent acute pancreatitis, fistulas, or persistent systemic inflammatory response syndrome, drainage may be necessary [11]. If drainage proves inadequate, necrosectomy is advised [12,13].

This study was conducted to evaluate the effectiveness and safety of endoscopic treatment in the management of WON and to assess combining percutaneous intervention in cases where endoscopic management was inadequate in a tertiary care center.

## 2. Materials and Methods

### 2.1. Patients and Pre-Procedure Evaluation

In the retrospective analysis of our prospective database, consecutive cases of endoscopic cystogastrostomy for WON between February 2019 and December 2023 were included in the study. All patients underwent preprocedural endoscopic evaluation. Additionally, the presence of vascular structures between the cavity and the lumen was assessed using computed tomography (CT) imaging. If vascular structures were observed or could not be clearly evaluated, the assessment was conducted using an echoendoscope (EUS) (Olympus GF-UE160 echoendoscope, Olympus, Tokyo, Japan). Patients without capsule integrity of the cavity, those with a distance of more than 10 mm between the lumen and the cavity, and individuals with visible vessels at the site where the cavity contacted the lumen were excluded.

Data collected from patients included demographic information, etiology, clinical manifestations, and results of imaging studies prior to the procedure. Furthermore, we documented the findings, results, and complications of our approach.

### 2.2. Definition

Walled-off necrosis was defined according to the revised Atlanta Classification [2]. Technical success was defined as the cannulation of the cavity, successful placement of a lumen-apposing metallic stent (LAMS) (Micro-Tech Co., Ltd., Nanjing, China) in the cavity, and successful necrosectomy if necessary. Clinical success was defined as the complete normalization of disease-related findings in clinical, laboratory, and CT imaging, or a reduction in WON size to 2 cm or a decrease in size by 25%. Recurrence was defined as the reappearance of fluid collection or necrosis after resolution. Complications such as bleeding, perforation, multi-organ failure, stent blockage, infection, and migration were documented. Complications were graded according to the AGREE classification [14].

### 2.3. Procedure

**Figure 1:**
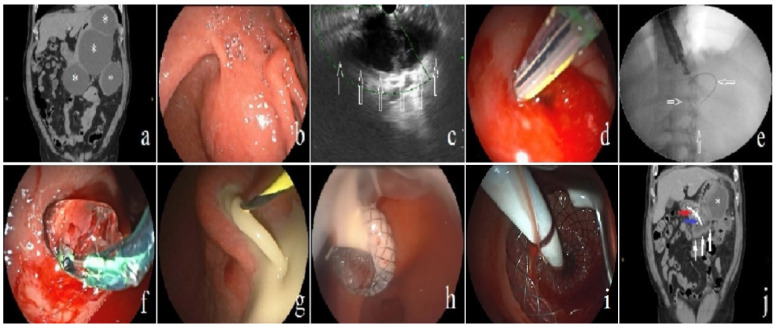
Placement of lumen-apposing metallic stent (LAMS) in the walled-off necrosis (WON) cavity. (**a**) CT demonstrating multi-lobulated WON cavities (asterisk). (**b**) Endoscopy demonstrating bulging of the gastric lumen. (**c**) Determining the WON cavity and selecting an appropriate drainage site with echoendoscope in a patient exhibiting no signs of bulging on the intestinal lumen (arrow). (**d**) Cavity puncture was performed with a needle knife sphincterotome. (**e**) Subsequently, a guidewire was carefully introduced into the cavity to facilitate further procedures (arrow). (**f**) Balloon dilatation was performed on the cystogastrostomy tract. (**g**) Purulent fluid was seen flowing into the lumen. (**h**) LAMS was placed to extend into the WON cavity. (**i**) To prevent migration, a double pigtail stent was positioned through the LAMS, extending into the cavity. (**j**) Three days post-procedure, the CT demonstrated the LAMS (red arrow), double pigtail stent (blue arrow), a visibly reduced volume of the drained WON cavity (white arrow), and another WON cavity (asterixis) that remained unaffected by endoscopic drainage.

All endoscopic procedures were performed using carbon dioxide insufflation by an experienced endoscopist under moderate sedation. Broad-spectrum antibiotics were administered to all patients before and after the procedure. A nasogastric tube was placed into the gastric lumen before the procedure, and fluid aspiration post-dilation was performed to prevent respiratory aspiration. In cases of luminal compression, the therapeutic duodenoscope (Fujinon ED-530XT Flexible Video Duodenoscope, Fujifilm Medical Co., Saitama, Japan) was utilized to identify the area of maximum pressure. Subsequently, the identified area was punctured with a needle knife sphincterotome as a conventional transmural drainage method. In patients without significant luminal compression, a 19-gauge fine needle aspiration needle was used to puncture the area determined as the safest location with an EUS. Afterward, the guidewire was advanced into the cavity. Cystogastrostomy tract dilation with a 6 or 8 mm dilatation balloon was performed to facilitate stent placement at the discretion of the endoscopist. This decision was based on factors such as the thickness of the collection wall and the density of the contents to be drained. After dilatation, a LAMS was deployed into the cavity under endoscopic and fluoroscopic guidance. To prevent LAMS migration, a double pigtail plastic stent (DPPS) was inserted through the LAMS (Figure 1). Patients with disconnected pancreatic duct syndrome (DPDS) identified during the imaging method were additionally subjected to pancreatic duct cannulation for transpapillary drainage. A pancreatic sphincterotomy was performed, and a pancreatic stent was placed to extend to the closest localization of duct disruption.

**Figure 2:**
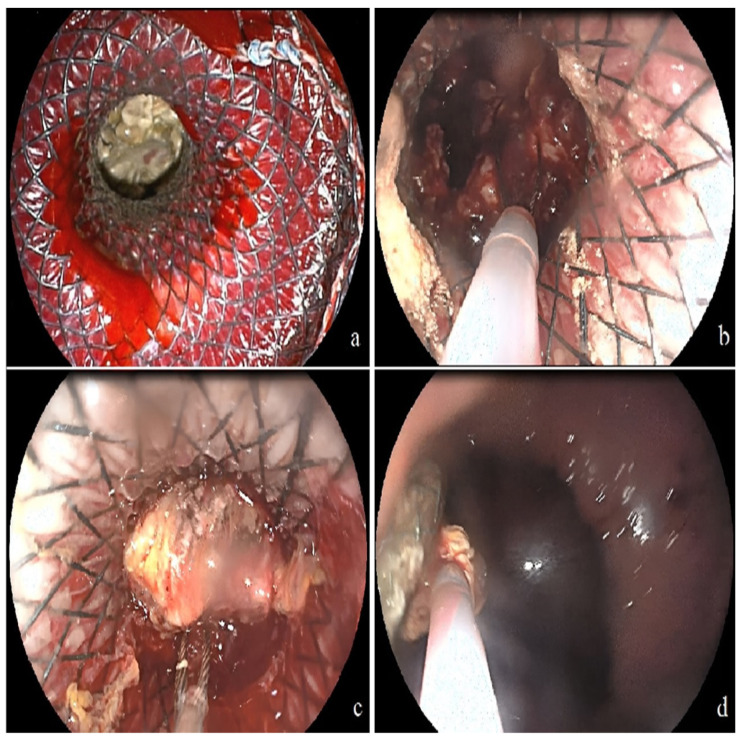
Direct endoscopic necrosectomy procedure. (**a**) Necrotic tissue in the walled-off necrosis cavity was seen through the lumen-apposing metallic stent (LAMS) with the endoscope. (**b**) The necrotic tissue was captured with a basket catheter. (**c**) The necrotic tissue was pulled through the LAMS into the gastric lumen. (**d**) Subsequently, the necrotic tissue was released into the gastric lumen.

**Figure 3:**
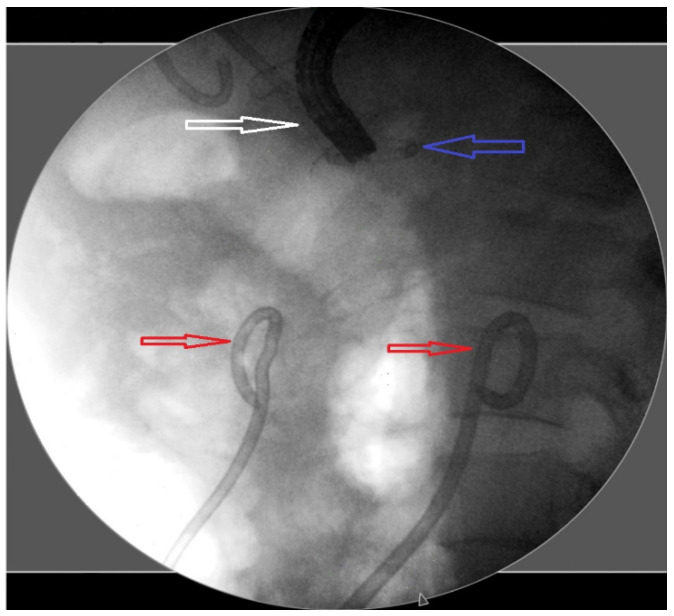
Image of a double pigtail stent with a lumen-apposing metallic stent and percutaneous drainage catheters on fluoroscopy. Figure 3 Fluoroscopy demonstrating the presence of the lumen-apposing metallic stent (white arrow) in the cystogastrostomy tract, along with the double pigtail stent (blue arrow) and two percutaneous drainage catheters (red arrows).

After the procedure, all patients followed up as inpatients and underwent clinical and laboratory examinations. If necessary, CT scans were performed, and the timing of follow-up procedures was decided on an individual basis. The decision of direct endoscopic necrosectomy (DEN) was made at the discretion of the physician based on insufficient resolution of necrosis. Necrosectomy was performed using a balloon, basket catheter, irrigation with sterile saline, and cytology brush (Figure 2). As a step-by-step approach, a percutaneous drainage catheter was placed in patients where clinical success could not be achieved after at least three successful endoscopic interventions (Figure 3). To expedite the resolution of necrosis and prevent stent and drain blockage, lavage was performed at least four times a day during subsequent follow-ups.

### 2.4. Post-Procedure Follow-Up

The stents were removed upon achieving clinical success, and patients were monitored with clinical, laboratory, and ultrasound examinations every three months for the first year. For patients with recurrent symptoms or documented recurrence of collections, CT imaging was repeated. In patients with clinical success, the total number of procedures was determined by including the last session in which the stent was removed.

### 2.5. Statistics

Statistical analysis was conducted using the 21st version of the Statistical Package for the Social Sciences (SPSS, IBM, Armonk, NY, USA). Continuous variables are presented as the median and interquartile range (IQR), while categorical data are presented as numbers and percentages. Due to the non-normal distribution of the data, outcomes between groups were compared using the Mann–Whitney U test for variance. The receiver operating characteristic (ROC) curve was utilized to calculate the area under the curve (AUC) for assessing the diagnostic value and accuracy of different parameters, with the best sensitivity and specificity determined by given cut-off values.

## 3. Results

### 3.1. Characteristics of Patients

A total of 11 patients (63.6% male, median age 48 years) with infected or complicated WON who underwent endoscopic intervention were included in this study. The causes underlying AP in these patients were gallstone (45.4%), alcohol (18.1%), acute-on-chronic pancreatitis (18.1%), and hypertriglyceridemia (9.0%). The cause of AP could not be determined in one (9.0%) patient. The median time interval between the diagnosis of AP attack and the diagnosis of WON was 54 days (IQR, 30 to 240 days). Abdominal pain was present in all patients, abdominal distension in 54.5%, early satiety in 54.5%, vomiting in 18.1%, and fever in 9.0% of cases. In laboratory examinations, white blood cell counts were above the upper limit of normal (ULN) in two (18%) patients, and amylase and/or lipase levels were above the ULN in eight (73%). Carcinoembryonic antigen and CA 19-9 levels were within normal limits in all patients. Diagnosis of WON was confirmed in all patients through abdominal CT scans. The median largest dimension of the WON was 150 mm (IQR, 92 to 238 mm). Single WON cavities were present in seven (63.6%) patients, while multiple cavities in four (36.3%) patients. Among these patients, the main location of WON involved the body and tail in six (54.5%) patients, the entire pancreas in two (18.1%), the body alone in one (9.0%), and the tail alone in one (9.0%). Additionally, in one (9.0%) patient, the cavity was located outside the pancreas. DPDS was detected in five (45.4%) patients, and gastric lumen bulging was observed in six (54.5%) patients during imaging examinations (Table 1).

### 3.2. Initial Outcomes of Endoscopic Intervention

Out of the 11 patients who underwent endoscopic intervention, 6 (54.5%) did so due to gastric outlet obstruction, 5 (45.4%) due to intractable pain, and 1 (9.0%) due to suspected infection. The conventional transmural drainage method was employed in six (54.5%) patients, while EUS-guided drainage was utilized in five (45.4%) patients. Successful cannulation of the cavity was achieved in all patients with the first puncture attempt. The cystogastrostomy tract was dilated in nine (81.8%) patients. LAMS with DPPS was placed into the WON in all patients. DEN was performed in seven (63.6%) patients. Additionally, transpapillary drainage was performed in five (45.4%) patients for DPDS. Surgical treatment was required in one (9.0%) patient due to perforation that occurred during the initial procedure. Technical success was 90.9%, while clinical success with only endoscopic treatment methods was 36.3% (Table 2).

### 3.3. The Addition of Percutaneous Intervention as a Step-Up Approach

In six (54.5%) patients, clinical success could not be achieved with three sessions of endoscopic management alone. Consequently, a step-up approach was adopted by inserting a percutaneous drainage catheter into the WON cavity. Both technical and clinical success were attained in all patients with a combination of percutaneous and endoscopic management (Table 2).

Patients who achieved clinical success with only endoscopic management had a median cavity diameter of 100–110 mm (IQR, 92 to 120 mm), whereas those who succeeded with a combined treatment method had a median cavity diameter of 200 mm (IQR, 130 to 238 mm) (*p* = 0.034). The cut-off value for endoscopic treatment success based on cavity diameter was 125 mm (AUC = 1.00; 95% CI: 1.00–1.00; *p* = 0.011). Additionally, all patients who achieved clinical success with endoscopic management alone had a single cavity, whereas among those successfully treated with a combination treatment method, four had two or more cavities (Table 2).

### 3.4. Outcomes of Complications, Recurrence, and Follow-Up

A total of 53 procedures were performed, with a median of five procedures (IQR, one to nine). Among the patients, two (18.2%) experienced major complications, with one (9.0%) encountering perforation and the other (9.0%) suffering from major bleeding necessitating replacement. In perforated patients, EUS-guided cannulation of the WON cavity was followed by balloon dilatation of the cystogastrostomy tract; however, despite the placement of LAMS, surgical treatment was ultimately necessary. The patient died due to multi-organ failure during the post-operative follow-up. Additionally, minor complications were observed in six patients, including LAMS migration and minor bleeding in two patients, LAMS migration and procedure-related infection in one patient, LAMS migration and blockage in one patient, and LAMS migration in two patients. LAMS migration was observed in those who were considered sufficiently drained with only LAMS without the need for DPPS during subsequent procedures. Out of the six patients, LAMS migration occurred into the gastric lumen in four patients, while it migrated into the cavity in two patients. In the two cases where LAMS migrated into the cavity, it was retrieved endoscopically through the cystogastrostomy tract, captured with a snare, removed, and then replaced. In two out of the four patients in whom LAMS migrated into the stomach lumen, the size of the WON cavity had significantly reduced, indicating no remaining necrosis. In these cases, only a DPPS extending into the cavity was placed through the cystogastrostomy tract. In the other two patients, no additional procedure was necessary as clinical success had already been achieved. Additionally, no complications related to stent infection or acute pancreatitis were detected after drainage or at the 1-month follow-up.

No major complications, such as organ perforation or major bleeding, related to the placement of percutaneous drainage catheter occurred in any of the patients. However, minor complications occurred in three (50%) patients. All of these were related to catheter occlusion, requiring either drain replacement or the use of a guide to clear the blockage.

The median length of hospital stay was 32 (IQR, 14 to 58) days, with a median follow-up time of 94 (IQR, 22 to 1105) days. Among the 10 patients who achieved clinical remission with a step-up treatment approach, recurrence occurred in only 1 patient during follow-up (Table 3).

## 4. Discussion

The incidence of acute necrotizing pancreatitis is increasing, and it can be a disease associated with a mortality rate ranging from 20% to 80%, attributed to septic complications and multiple organ failure [15]. Endoscopic treatment methods have been reported to achieve high success rates with low morbidity and mortality rates, along with offering lower total costs and improved quality of life [4,16,17]. Additionally, the choice of stent for transmural drainage in endoscopic management is a current area of debate. A recent review highlighted the use of LAMS in patients with WON, reporting a resolution rate of 87.7% with transmural drainage using LAMS. Complications included stent occlusion (7.5%), bleeding (6.2%), perforation (3.8%), migration (7.8%), and occlusion (11.7%) [18]. Consequently, while surgery has traditionally been the predominant approach, minimally invasive methods, particularly endoscopic treatments, are increasingly favored as the initial step [19,20,21].

We included 11 consecutive patients with infected or complicated WON who were referred to our tertiary referral center over a 4-year period. Technical success was achieved in 90.9% despite the development of retroperitoneal perforation in one patient, while clinical success with endoscopic treatment alone was 36.3%. An additional percutaneous drainage catheter was placed in patients where an adequate response could not be achieved. Clinical success was attained in all patients who underwent this combination treatment method. During a total of 53 procedures, major complications occurred in 18.1% of patients, while minor complications occurred in 6% of patients. Among all complications, LAMS migration (54.5%) was the most common, and all of these occurred after the decision not to place DPPS in the follow-up procedures. Our clinical success and major complication results were comparable to previous studies (20–25).

In our study, the clinical success achieved through endoscopic management alone was relatively low (36.3%), despite achieving high technical success rates, transpapillary drainage in all patients with DPDS, the placement of LAMS in all patients to facilitate drainage, and DEN in indicated cases. We attribute this low success rate of endoscopic treatment alone compared to previous studies to several factors, including the significantly large average cavity diameter, the presence of multiple unrelated cavities in four patients, and the complexity of cases typically referred to our center as a tertiary referral center. Out of our patient cohort, only one individual encountered a procedure-related perforation, which occurred during cannulation utilizing the EUS-guided method. The absence of perforations in patients undergoing cavity cannulation via conventional transmural drainage in our study can be attributed to several factors. These include thorough preprocedural evaluation of patients radiologically and endoscopically, the performance of cystogastrostomy tract dilation in deemed necessary cases (the fluidity of the drainage fluid was the main factor), the use of LAMS in all patients, and the fact that an experienced endoscopist conducted all procedures. The recent systematic review and meta-analysis by Bang et al. evaluated the use of LAMS and plastic stent, reporting no significant difference in clinical outcomes except for procedure duration [22]. Since our study did not include a patient group with only plastic stent placement for the drainage of WON, we could not make a comparison. However, we believe that there are additional benefits to using LAMS. The most significant of these is the closure of gaps without causing a clinical outcome in minor perforation cases that we cannot detect during the procedure. Another benefit is facilitating entry into the cavity during necrosectomy procedures. In cases where adequate necrosis resolution could not be achieved, additional procedures were required for cavity washing. In patients with an additional percutaneous drainage catheter, providing drainage to the intestinal lumen through external washing with a low risk of blockage is also a valuable contribution. Additionally, washing could be easily performed within the day without the need for repeated procedures. Mohan et al. reported a migration rate of 5.1% for LAMS [23]. Our migration rate for LAMS was 11.3% in a total of 53 procedures. All migrations occurred in follow-up procedures after procedures where DPPS was not placed. It is clear that placing DPPS in every session where LAMS will be left in place will reduce migration rates. Additionally, re-cavity cannulation after migration was quite easy in our patients in whom LAMS was placed before, and this can be considered an additional advantage of using LAMS.

We achieved clinical success with the combination of endoscopic and percutaneous treatment, as a step-up therapeutic approach, in all six patients for whom clinical success could not be achieved with three sessions of endoscopic management alone. We believe there are several main reasons for this high success rate. Firstly, the addition of percutaneous drainage to the treatment allows for the drainage of cavities that cannot be drained endoscopically. Secondly, the reduction in the size of large cavities through endoscopic drainage may lead to the development of collapsed regions within the cavity, impeding effective drainage by endoscopic modalities alone. The addition of percutaneous drainage enables the drainage of regions distant from the cystogastrostomy tract, which may be difficult to access solely through endoscopic means. Additionally, frequent cavity washouts within the day help to maintain optimal drainage and promote the resolution of necrotic tissue, contributing to improved patient outcomes. In our study, we conducted a comparative analysis between patients who achieved successful outcomes with endoscopic management alone and those who required a combined treatment approach for success. The findings suggest that in instances involving multiple cavities or cavities of significant size (≥125 mm), relying solely on endoscopic management may prove insufficient. In such cases, a combined treatment approach emerges as an effective strategy.

According to the recent results of 117 patients with WON treated with a percutaneous drainage catheter reported by Mallick et al., complications included external pancreatic fistula in 34.2% of patients, drain blockage in 14.5%, drain slippage in 11.1%, and bleeding in 5.1%. Among those with an external pancreatic fistula, 80% required the placement of a pancreatic stent. Additionally, 12% of patients required surgery, and mortality occurred in 13.7% [24]. None of our patients experienced major complications such as organ perforation or significant bleeding during the percutaneous interventions. A total of 50% of our patients with combining percutaneous modalities experienced drain obstruction. No instances of external pancreatic fistula, drain slippage, or bleeding were observed. None of the patients required surgery due to percutaneous intervention, and there was no mortality. We believe that the placement of a pancreatic stent before percutaneous intervention in patients with pre-existing DPDS, along with meticulous follow-up to prevent drain slippage, was effective.

The strength of this study lies in its inclusion of patients treated with a pre-defined step-up strategy and the performance of all procedures by an experienced endoscopist. However, our study has several limitations. Firstly, it is retrospective in design; however, we believe that this limitation is largely overcome by the pre-defined treatment strategy and the retrospective analysis of our prospective database. Secondly, another limitation is the number of included patients. This can be attributed to the fact that our clinic is a tertiary referral center. Therefore, severe cases that cannot be adequately managed in other healthcare institutions are often referred to our hospital. We anticipate that addressing this issue in future multi-center studies or meta-analyses may provide a more comprehensive perspective. Another limitation is that EUS-guided interventions were not performed in all patients. However, no major complications occurred in patients where we utilized the conventional transmural drainage method for interventions. Therefore, we are confident that this limitation did not significantly impact our results.

In conclusion, endoscopic management is recommended as the primary treatment method for WON due to its less invasive nature and higher safety profile. However, in cases where there are large (≥125 mm) or multi-lobulated WON cavities and clinical success cannot be achieved with three interventions, the inclusion of percutaneous drainage in the treatment is highly successful and safe. Ultimately, it can minimize the need for surgery, which is a more invasive treatment modality.

## Figures and Tables

**Table 1:** Characteristics of patients with walled-off necrosis who underwent endoscopic management.

	Total *n* (%)
**Age (median years)**	48 (24–61)
**Sex**	
Male	7 (63.6)
Female	4 (36.3)
**Cause of pancreatitis**	
Alcohol	2 (18.1)
Gallstone	5 (45.4)
Acute or chronic pancreatitis	2 (18.1)
Hypertriglyceridemia	1 (9.0)
Idiopathic	1 (9.0)
**Diagnosis time from pancreatitis to WON (median days)**	54 (30–240)
**Clinical presentation**	
Abdominal pain	11 (100)
Vomiting	2 (18.1)
Fever	1 (9.0)
Abdominal distention	6 (54.5)
Early satiety	6 (54.5)
**Laboratory investigation**	
Elevated WBC	2 (18.1)
Elevated amylase and lipase	8 (72.7)
Normal CEA and Ca 19-9	11 (100)
**Diagnosis by imaging**	
Abdominal CT or MRI	11 (100)
**Largest dimension of WON cavity (mm)**	150 (92–238)
**No. of WON cavities**	
Single	7 (63.6)
Multiple	4 (36.3)
**Main location of WON**	
Body	1 (9.0)
Tail	1 (9.0)
Body and tail	6 (54.5)
Entire pancreas	2 (18.1)
Extrapancreatic location	1 (9.0)
**Presence of DPD**	5 (45.4)
**Presence of bulging into the stomach**	6 (54.5)
**Intra-cavity echogenicity**	
Anechogenic	11 (100)

Data are presented as median (IQR) or frequency (%). WON, walled-off necrosis. DPD, disconnected pancreatic duct. WBC, white blood cell. CEA, carcinoembryonic antigen. CT, computed tomography. MRI, magnetic resonance imaging.

**Table 2:** Procedural details and outcomes of a step-up approach in patients with walled-off necrosis.

	Total *n* (%)
**Indication of intervention**	
Suspected infection	1 (9.0)
Intractable pain	5 (45.4)
Gastric outlet obstruction	6 (54.5)
**Preprocedural intravenous antibiotics**	11 (100)
**Drainage method**	
CTD	6 (54.5)
EUS	5 (45.4)
Additional transpapillary drainage	5 (45.4)
**Route**	
Transgastric	11 (100)
Transduodenal	0 (0)
**Insufflation during the procedure**	
Room air	0 (0)
CO_2_ gas	11 (100)
**Cavity cannulation success at first attempt**	11 (100)
**Tract dilatation**	9 (81.8)
**Types of stents**	
LAMS with DPPS	11 (100)
**Size of LAMS**	
16 × 15 mm	5 (45.4)
16 × 20 mm	6 (54.5)
**Direct endoscopic necrosectomy needed**	7 (63.6)
**Additional percutaneous intervention**	6 (54.5)
**Surgery treatment needed**	1 (9.0)
**Technical success**	10 (90.9)
**Clinical success with only endoscopic management**	4 (36.3)
Largest dimension of WON cavity (mm)	100–110 (92–120)
Single WON cavity	4 (100)
Multiple WON cavities	0 (0)
**Clinical success with a combination of percutaneous and endoscopic management**	6 (54.5)
Largest dimension of WON cavity (mm)	200 (130–238)
Single WON cavity	2 (33.3)
Multiple WON cavities	4 (66.6)
**Clinical failure**	1 (9.0)

Data are presented as median (IQR) or frequency (%). CTD, conventional transmural drainage. EUS, endoscopic ultrasonography. CO_2_, carbon dioxide. LAMS, lumen-apposing metallic stent. DPPS, double pigtail plastic stent.

**Table 3:** Outcomes of complications, recurrence, and follow-up.

	Total *n* (%)
**No. of procedures**	5 (1–9)
**Complications of endoscopic management**	
**Major complications**	2 (18.1)
Major bleeding	1 (9.0)
Perforation	1 (9.0)
**Minor complications**	6 (54.5)
Minor bleeding	2 (18.1)
LAMS blockage	1 (9.0)
Procedure-related infection	1 (9.0)
LAMS migration into the lumen	4 (36.3)
LAMS migration into the cavity	2 (18.1)
**Complications of percutaneous intervention**	
Blockage	3 (50)
**Recurrence**	1 (9.0)
**Mortality**	1 (9.0)
**Hospital stay (median days)**	32 (14–58)
**Total follow-up (median days)**	123 (55–1125)

Data are presented as median (IQR) or frequency (%). LAMS, lumen-apposing metallic stent.

## Data Availability

The data used and analyzed in the current study are available from the corresponding author upon reasonable request.
